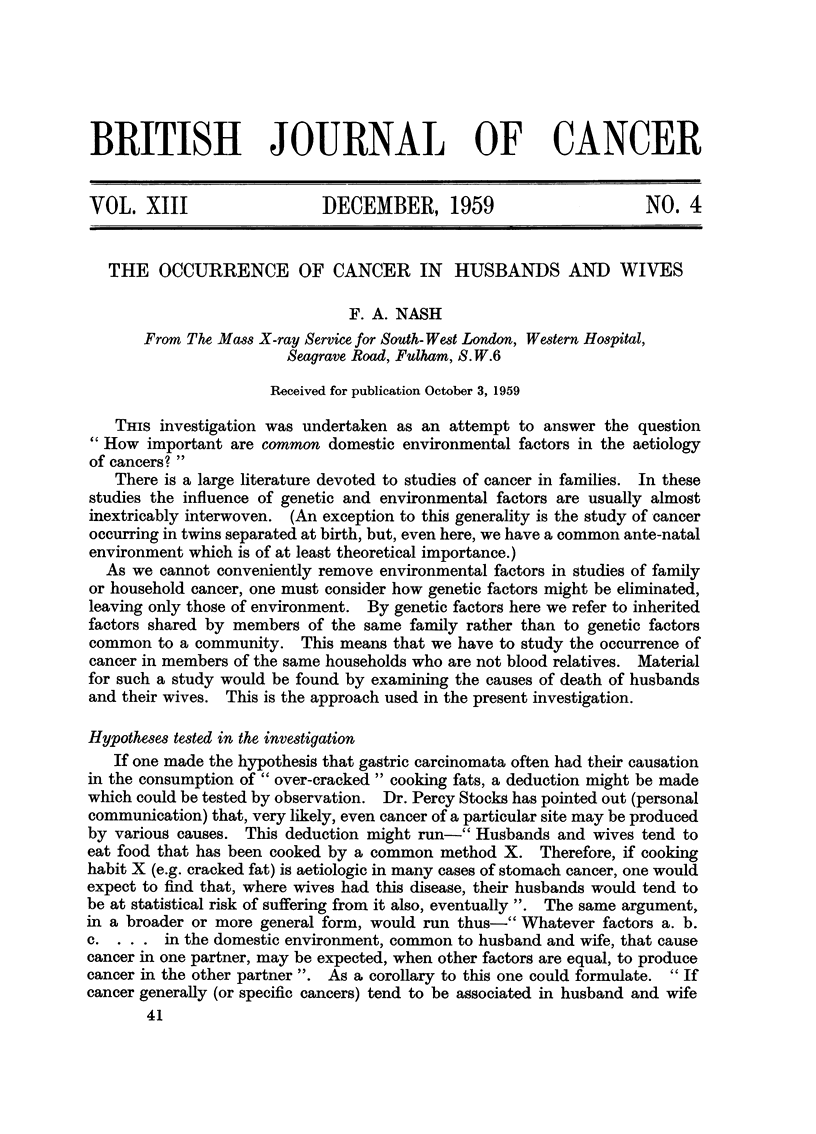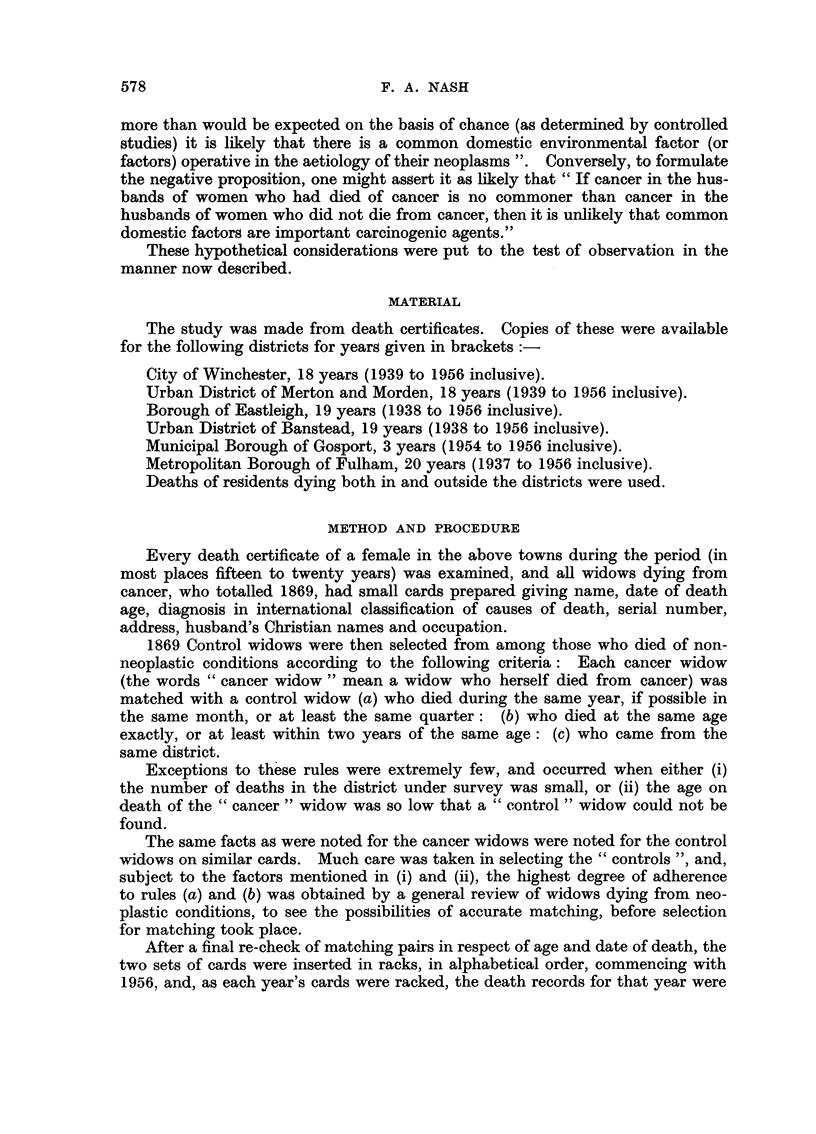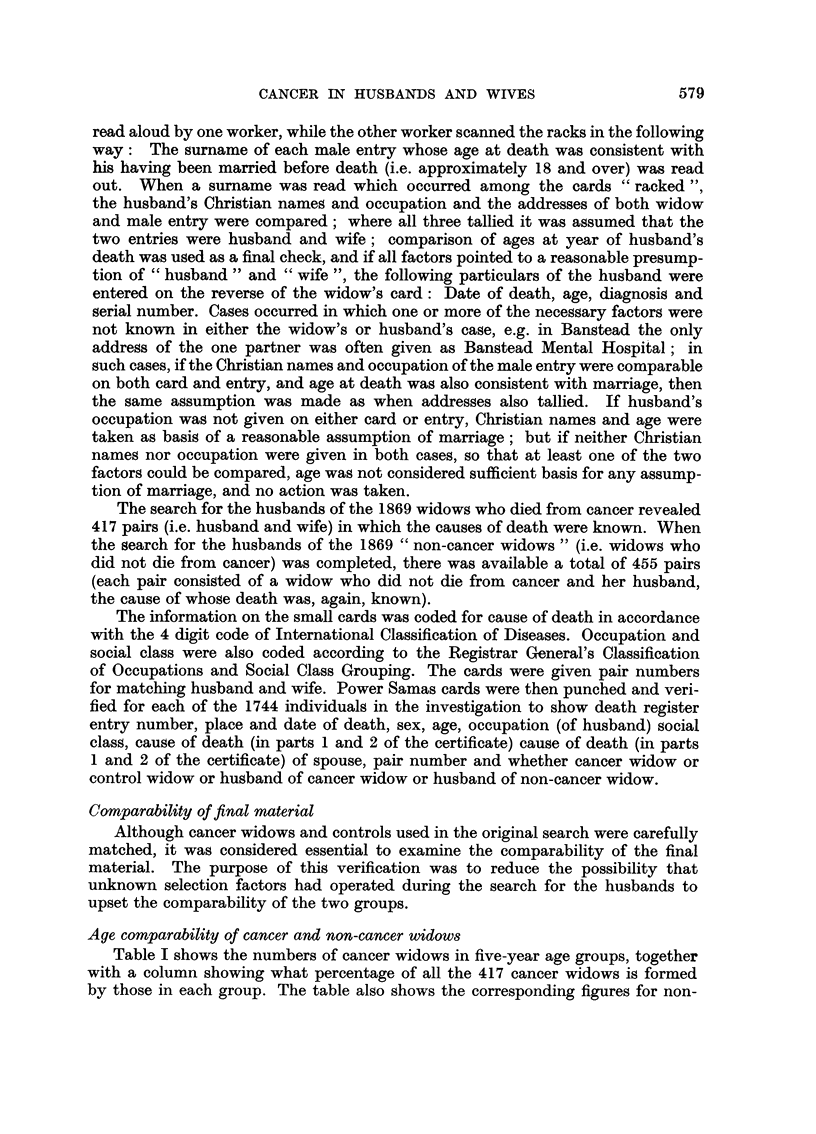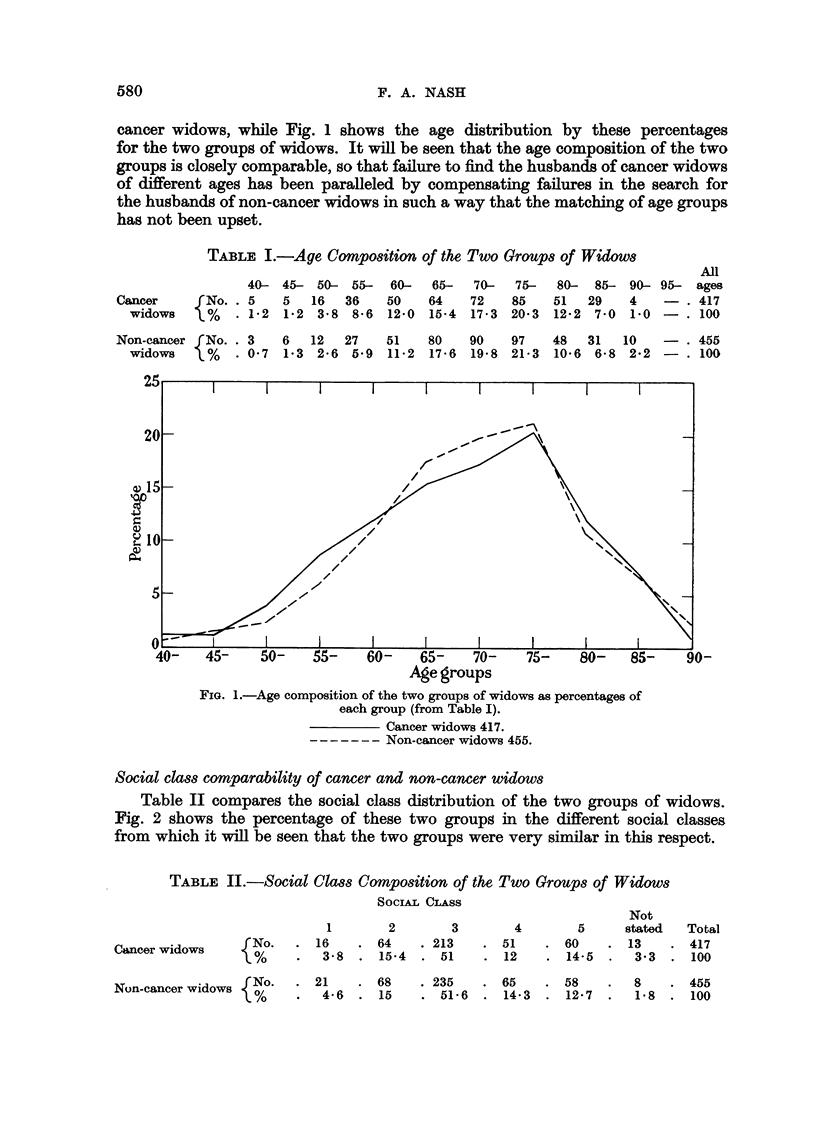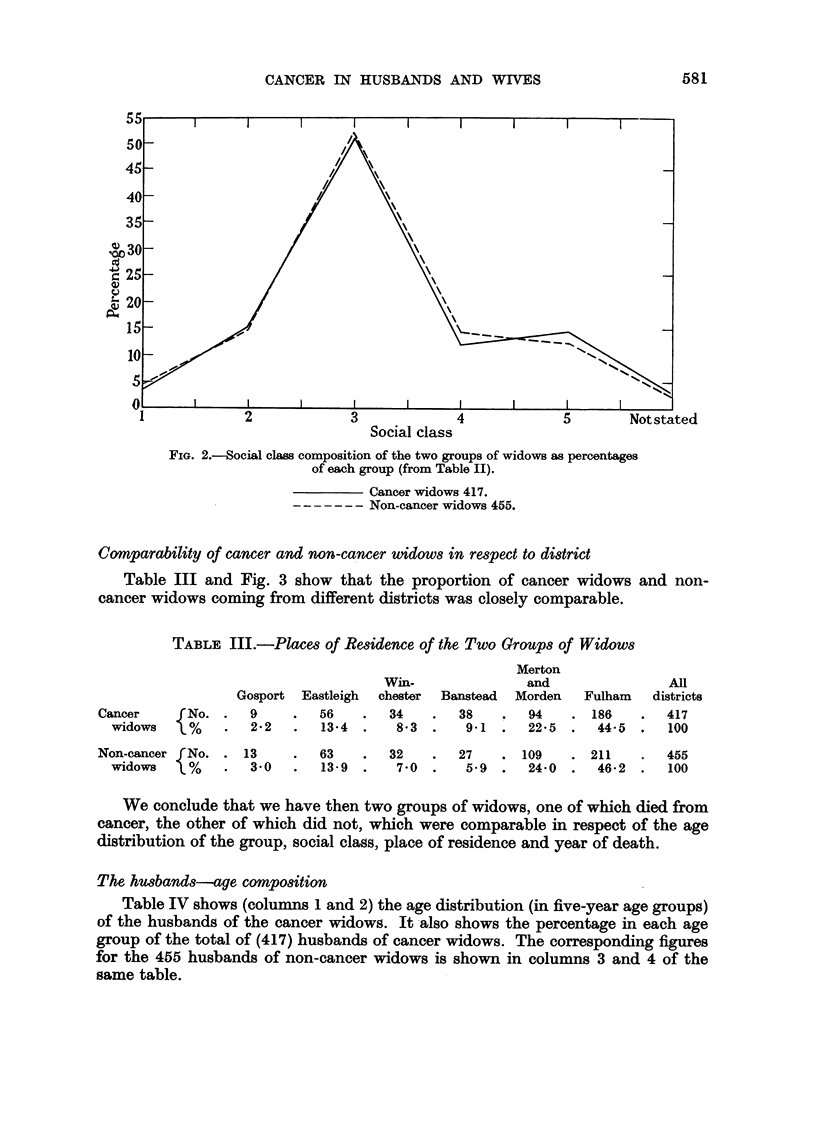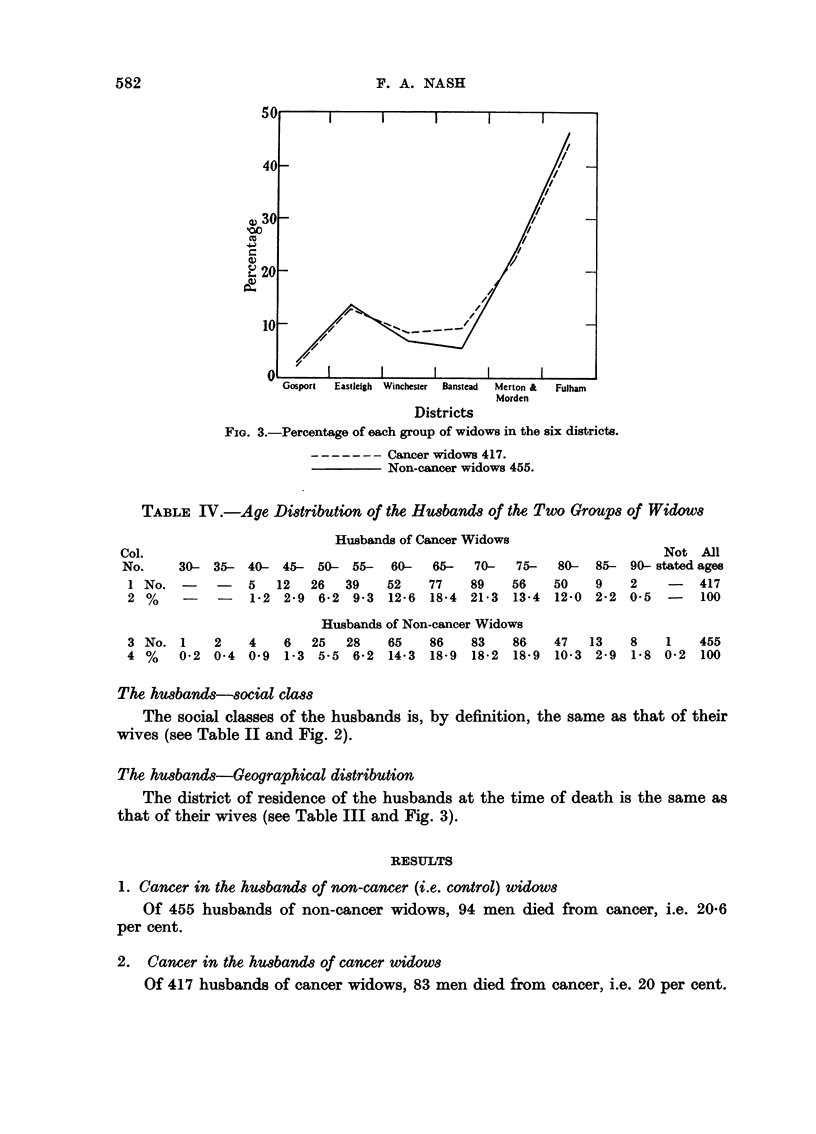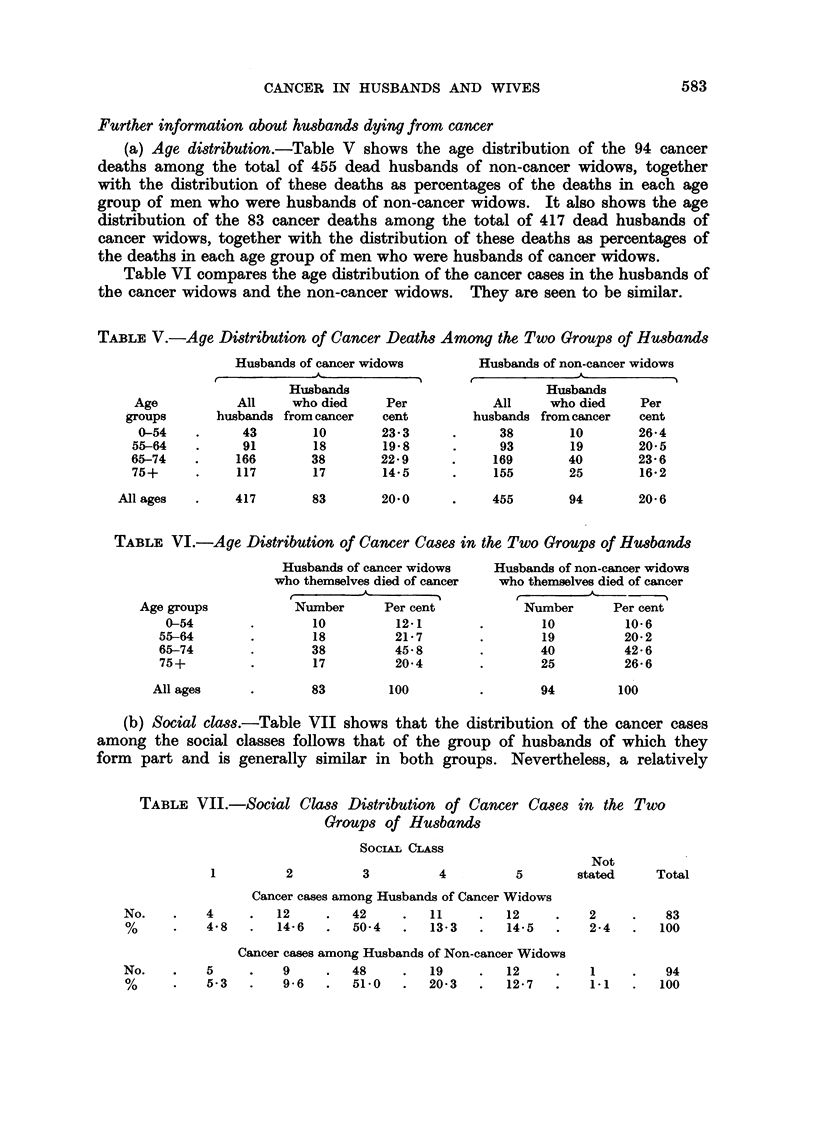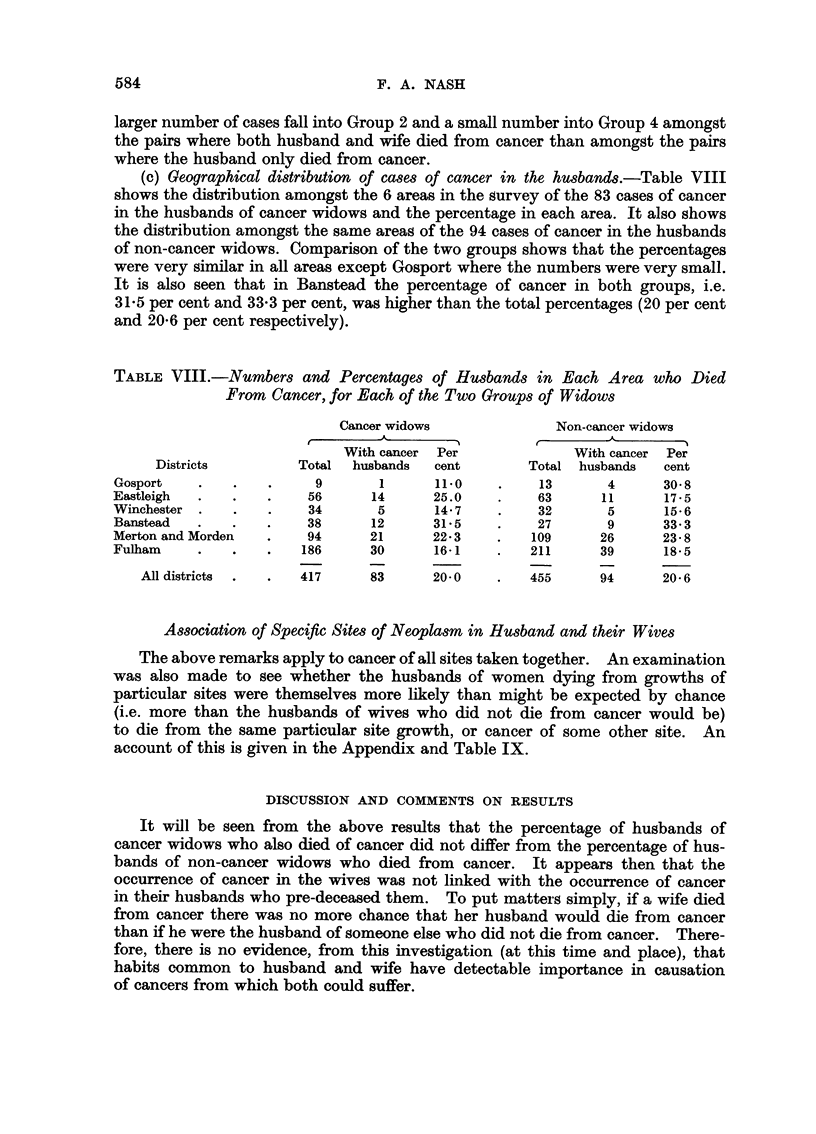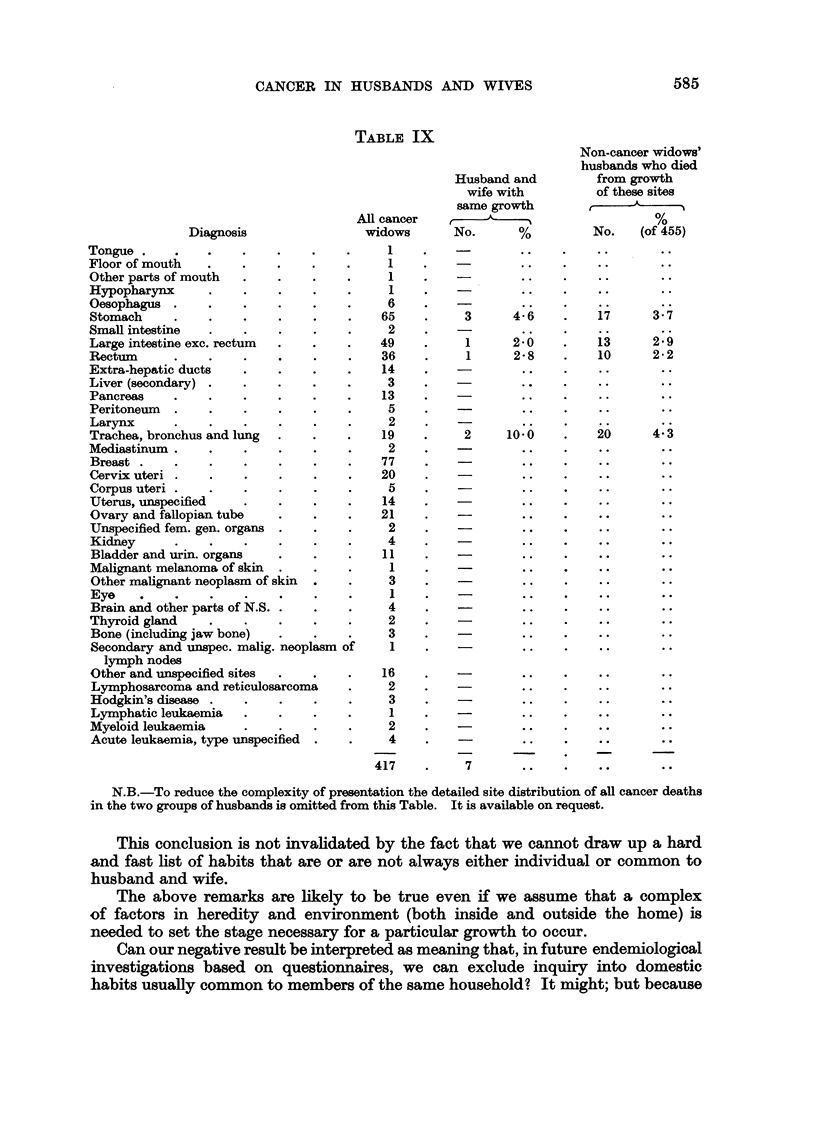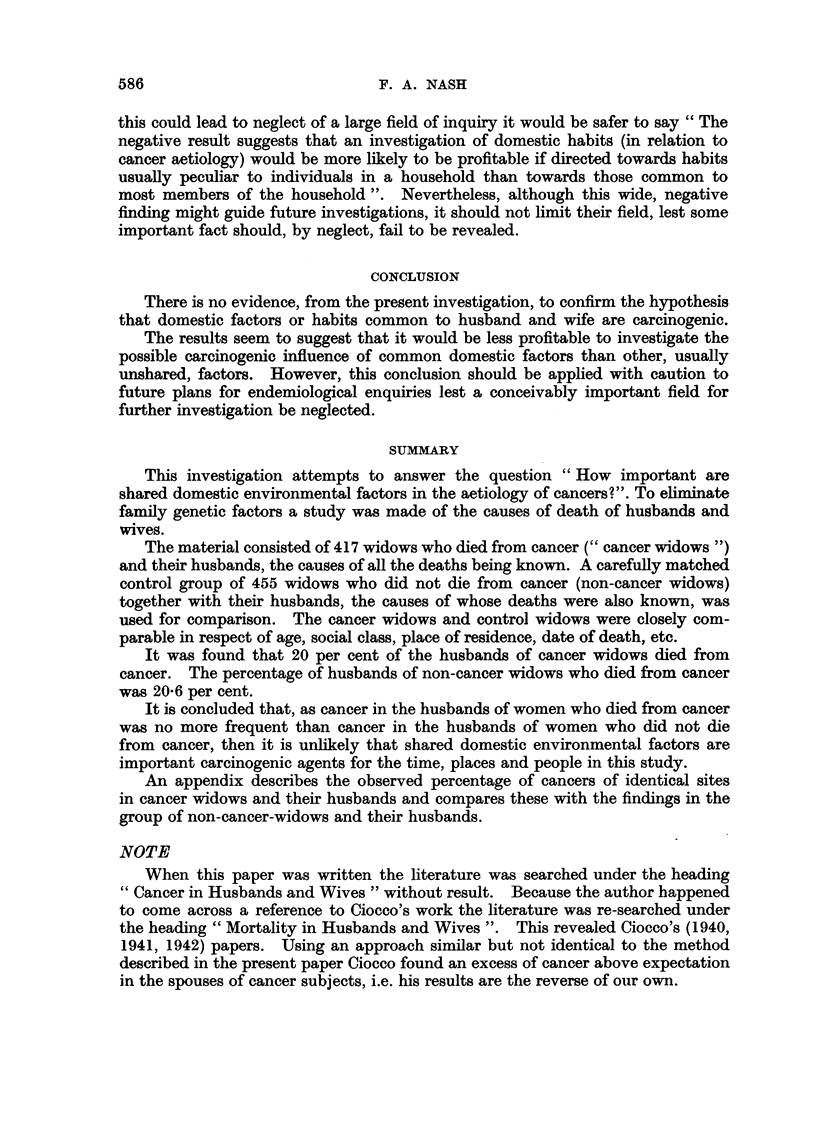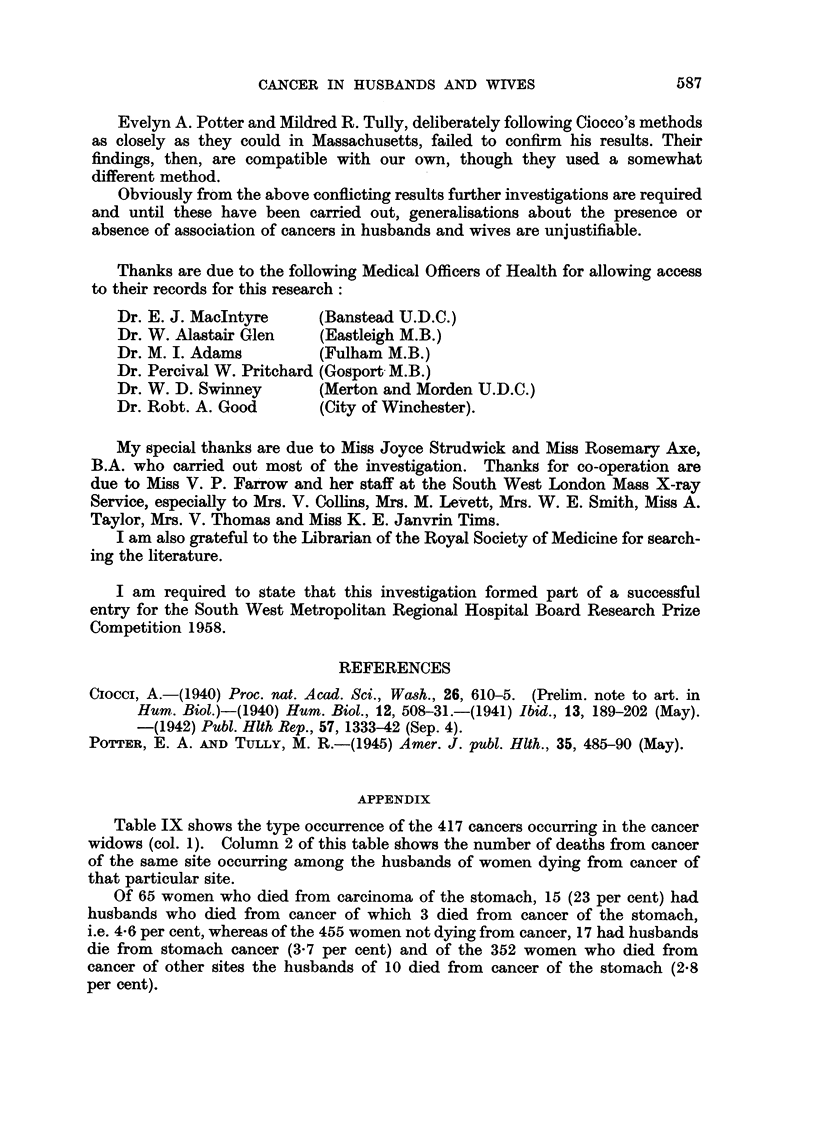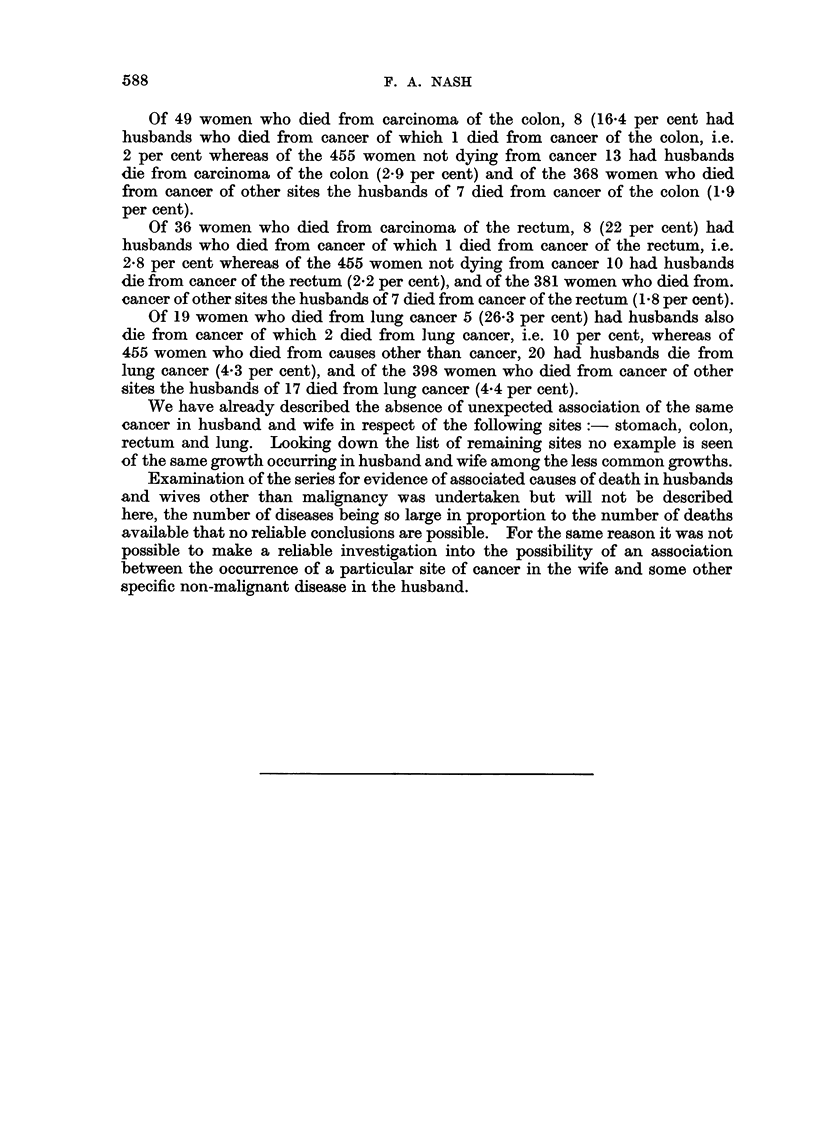# The Occurrence of Cancer in Husbands and Wives

**DOI:** 10.1038/bjc.1959.62

**Published:** 1959-12

**Authors:** F. A. Nash


### BRITISH JOURNAL OF CANCER

VOL. XIII DECEMBER, 1959 NO. 4

### THE OCCURRENCE OF CANCER IN HUSBANDS AND WIVES

F. A. NASH

*From The Mass X-ray Service for South-West London, Western Hospital, Seagrave Road, Fulham, S.W.6*

Received for publication October 3, 1959

THIS investigation was undertaken as an attempt to answer the question " How important are *common* domestic environmental factors in the aetiology of cancers? "

There is a large literature devoted to studies of cancer in families. In these studies the influence of genetic and environmental factors are usually almost inextricably interwoven. (An exception to this generality is the study of cancer occurring in twins separated at birth, but, even here, we have a common ante-natal environment which is of at least theoretical importance.)

As we cannot conveniently remove environmental factors in studies of family or household cancer, one must consider how genetic factors might be eliminated, leaving only those of environment. By genetic factors here we refer to inherited factors shared by members of the same family rather than to genetic factors common to a community. This means that we have to study the occurrence of cancer in members of the same households who are not blood relatives. Material for such a study would be found by examining the causes of death of husbands and their wives. This is the approach used in the present investigation.

*Hypotheses tested in the investigation*

If one made the hypothesis that gastric carcinomata often had their causation in the consumption of " over-cracked " cooking fats, a deduction might be made which could be tested by observation. Dr. Percy Stocks has pointed out (personal communication) that, very likely, even cancer of a particular site may be produced by various causes. This deduction might run—" Husbands and wives tend to eat food that has been cooked by a common method X. Therefore, if cooking habit X (e.g. cracked fat) is aetiologic in many cases of stomach cancer, one would expect to find that, where wives had this disease, their husbands would tend to be at statistical risk of suffering from it also, eventually ". The same argument, in a broader or more general form, would run thus—" Whatever factors a. b. c. . . . in the domestic environment, common to husband and wife, that cause cancer in one partner, may be expected, when other factors are equal, to produce cancer in the other partner ". As a corollary to this one could formulate. " If cancer generally (or specific cancers) tend to be associated in husband and wife more than would be expected on the basis of chance (as determined by controlled studies) it is likely that there is a common domestic environmental factor (or factors) operative in the aetiology of their neoplasms ". Conversely, to formulate the negative proposition, one might assert it as likely that " If cancer in the husbands of women who had died of cancer is no commoner than cancer in the husbands of women who did not die from cancer, then it is unlikely that common domestic factors are important carcinogenic agents."

These hypothetical considerations were put to the test of observation in the manner now described.

### MATERIAL

The study was made from death certificates. Copies of these were available for the following districts for years given in brackets :—

City of Winchester, 18 years (1939 to 1956 inclusive).
Urban District of Merton and Morden, 18 years (1939 to 1956 inclusive).
Borough of Eastleigh, 19 years (1938 to 1956 inclusive).
Urban District of Banstead, 19 years (1938 to 1956 inclusive).
Municipal Borough of Gosport, 3 years (1954 to 1956 inclusive).
Metropolitan Borough of Fulham, 20 years (1937 to 1956 inclusive).
Deaths of residents dying both in and outside the districts were used.

### METHOD AND PROCEDURE

Every death certificate of a female in the above towns during the period (in most places fifteen to twenty years) was examined, and all widows dying from cancer, who totalled 1869, had small cards prepared giving name, date of death age, diagnosis in international classification of causes of death, serial number, address, husband's Christian names and occupation.

1869 Control widows were then selected from among those who died of non-neoplastic conditions according to the following criteria : Each cancer widow (the words " cancer widow " mean a widow who herself died from cancer) was matched with a control widow (*a*) who died during the same year, if possible in the same month, or at least the same quarter : (*b*) who died at the same age exactly, or at least within two years of the same age : (*c*) who came from the same district.

Exceptions to these rules were extremely few, and occurred when either (i) the number of deaths in the district under survey was small, or (ii) the age on death of the " cancer " widow was so low that a " control " widow could not be found.

The same facts as were noted for the cancer widows were noted for the control widows on similar cards. Much care was taken in selecting the " controls ", and, subject to the factors mentioned in (i) and (ii), the highest degree of adherence to rules (*a*) and (*b*) was obtained by a general review of widows dying from neoplastic conditions, to see the possibilities of accurate matching, before selection for matching took place.

After a final re-check of matching pairs in respect of age and date of death, the two sets of cards were inserted in racks, in alphabetical order, commencing with 1956, and, as each year's cards were racked, the death records for that year were read aloud by one worker, while the other worker scanned the racks in the following way: The surname of each male entry whose age at death was consistent with his having been married before death (i.e. approximately 18 and over) was read out. When a surname was read which occurred among the cards "racked", the husband's Christian names and occupation and the addresses of both widow and male entry were compared; where all three tallied it was assumed that the two entries were husband and wife; comparison of ages at year of husband's death was used as a final check, and if all factors pointed to a reasonable presumption of "husband" and "wife", the following particulars of the husband were entered on the reverse of the widow's card: Date of death, age, diagnosis and serial number. Cases occurred in which one or more of the necessary factors were not known in either the widow's or husband's case, e.g. in Banstead the only address of the one partner was often given as Banstead Mental Hospital; in such cases, if the Christian names and occupation of the male entry were comparable on both card and entry, and age at death was also consistent with marriage, then the same assumption was made as when addresses also tallied. If husband's occupation was not given on either card or entry, Christian names and age were taken as basis of a reasonable assumption of marriage; but if neither Christian names nor occupation were given in both cases, so that at least one of the two factors could be compared, age was not considered sufficient basis for any assumption of marriage, and no action was taken.

The search for the husbands of the 1869 widows who died from cancer revealed 417 pairs (i.e. husband and wife) in which the causes of death were known. When the search for the husbands of the 1869 "non-cancer widows" (i.e. widows who did not die from cancer) was completed, there was available a total of 455 pairs (each pair consisted of a widow who did not die from cancer and her husband, the cause of whose death was, again, known).

The information on the small cards was coded for cause of death in accordance with the 4 digit code of International Classification of Diseases. Occupation and social class were also coded according to the Registrar General's Classification of Occupations and Social Class Grouping. The cards were given pair numbers for matching husband and wife. Power Samas cards were then punched and verified for each of the 1744 individuals in the investigation to show death register entry number, place and date of death, sex, age, occupation (of husband) social class, cause of death (in parts 1 and 2 of the certificate) cause of death (in parts 1 and 2 of the certificate) of spouse, pair number and whether cancer widow or control widow or husband of cancer widow or husband of non-cancer widow.

*Comparability of final material*

Although cancer widows and controls used in the original search were carefully matched, it was considered essential to examine the comparability of the final material. The purpose of this verification was to reduce the possibility that unknown selection factors had operated during the search for the husbands to upset the comparability of the two groups.

*Age comparability of cancer and non-cancer widows*

Table I shows the numbers of cancer widows in five-year age groups, together with a column showing what percentage of all the 417 cancer widows is formed by those in each group. The table also shows the corresponding figures for noncancer widows, while Fig. 1 shows the age distribution by these percentages for the two groups of widows. It will be seen that the age composition of the two groups is closely comparable, so that failure to find the husbands of cancer widows of different ages has been paralleled by compensating failures in the search for the husbands of non-cancer widows in such a way that the matching of age groups has not been upset.

TABLE I.—*Age Composition of the Two Groups of Widows*

| | | 40– | 45– | 50– | 55– | 60– | 65– | 70– | 75– | 80– | 85– | 90– | 95– | All ages |
|---|---|---|---|---|---|---|---|---|---|---|---|---|---|---|
| Cancer widows | No. | 5 | 5 | 16 | 36 | 50 | 64 | 72 | 85 | 51 | 29 | 4 | — | 417 |
| | % | 1·2 | 1·2 | 3·8 | 8·6 | 12·0 | 15·4 | 17·3 | 20·3 | 12·2 | 7·0 | 1·0 | — | 100 |
| Non-cancer widows | No. | 3 | 6 | 12 | 27 | 51 | 80 | 90 | 97 | 48 | 31 | 10 | — | 455 |
| | % | 0·7 | 1·3 | 2·6 | 5·9 | 11·2 | 17·6 | 19·8 | 21·3 | 10·6 | 6·8 | 2·2 | — | 100 |

FIG. 1.—Age composition of the two groups of widows as percentages of each group (from Table I).

———— Cancer widows 417.

- - - - - - - Non-cancer widows 455.

*Social class comparability of cancer and non-cancer widows*

Table II compares the social class distribution of the two groups of widows. Fig. 2 shows the percentage of these two groups in the different social classes from which it will be seen that the two groups were very similar in this respect.

TABLE II.—*Social Class Composition of the Two Groups of Widows*

| | | Social Class 1 | 2 | 3 | 4 | 5 | Not stated | Total |
|---|---|---|---|---|---|---|---|---|
| Cancer widows | No. | 16 | 64 | 213 | 51 | 60 | 13 | 417 |
| | % | 3·8 | 15·4 | 51 | 12 | 14·5 | 3·3 | 100 |
| Non-cancer widows | No. | 21 | 68 | 235 | 65 | 58 | 8 | 455 |
| | % | 4·6 | 15 | 51·6 | 14·3 | 12·7 | 1·8 | 100 |

FIG. 2.—Social class composition of the two groups of widows as percentages of each group (from Table II).

——— Cancer widows 417.
- - - - - - - Non-cancer widows 455.

*Comparability of cancer and non-cancer widows in respect to district*

Table III and Fig. 3 show that the proportion of cancer widows and non-cancer widows coming from different districts was closely comparable.

TABLE III.—*Places of Residence of the Two Groups of Widows*

| | | Gosport | Eastleigh | Win-chester | Banstead | Merton and Morden | Fulham | All districts |
|---|---|---|---|---|---|---|---|---|
| Cancer widows | No. | 9 | 56 | 34 | 38 | 94 | 186 | 417 |
| | % | 2·2 | 13·4 | 8·3 | 9·1 | 22·5 | 44·5 | 100 |
| Non-cancer widows | No. | 13 | 63 | 32 | 27 | 109 | 211 | 455 |
| | % | 3·0 | 13·9 | 7·0 | 5·9 | 24·0 | 46·2 | 100 |

We conclude that we have then two groups of widows, one of which died from cancer, the other of which did not, which were comparable in respect of the age distribution of the group, social class, place of residence and year of death.

*The husbands—age composition*

Table IV shows (columns 1 and 2) the age distribution (in five-year age groups) of the husbands of the cancer widows. It also shows the percentage in each age group of the total of (417) husbands of cancer widows. The corresponding figures for the 455 husbands of non-cancer widows is shown in columns 3 and 4 of the same table.

FIG. 3.—Percentage of each group of widows in the six districts.

------- Cancer widows 417.
———— Non-cancer widows 455.

TABLE IV.—*Age Distribution of the Husbands of the Two Groups of Widows*

| Col. No. | | 30– | 35– | 40– | 45– | 50– | 55– | 60– | 65– | 70– | 75– | 80– | 85– | 90– | Not stated | All ages |
|---|---|---|---|---|---|---|---|---|---|---|---|---|---|---|---|---|
| | Husbands of Cancer Widows | | | | | | | | | | | | | | | |
| 1 | No. | — | — | 5 | 12 | 26 | 39 | 52 | 77 | 89 | 56 | 50 | 9 | 2 | — | 417 |
| 2 | % | — | — | 1·2 | 2·9 | 6·2 | 9·3 | 12·6 | 18·4 | 21·3 | 13·4 | 12·0 | 2·2 | 0·5 | — | 100 |
| | Husbands of Non-cancer Widows | | | | | | | | | | | | | | | |
| 3 | No. | 1 | 2 | 4 | 6 | 25 | 28 | 65 | 86 | 83 | 86 | 47 | 13 | 8 | 1 | 455 |
| 4 | % | 0·2 | 0·4 | 0·9 | 1·3 | 5·5 | 6·2 | 14·3 | 18·9 | 18·2 | 18·9 | 10·3 | 2·9 | 1·8 | 0·2 | 100 |

*The husbands—social class*

The social classes of the husbands is, by definition, the same as that of their wives (see Table II and Fig. 2).

*The husbands—Geographical distribution*

The district of residence of the husbands at the time of death is the same as that of their wives (see Table III and Fig. 3).

### RESULTS

1. *Cancer in the husbands of non-cancer (i.e. control) widows*

Of 455 husbands of non-cancer widows, 94 men died from cancer, i.e. 20·6 per cent.

2. *Cancer in the husbands of cancer widows*

Of 417 husbands of cancer widows, 83 men died from cancer, i.e. 20 per cent.

*Further information about husbands dying from cancer*

(a) *Age distribution.*—Table V shows the age distribution of the 94 cancer deaths among the total of 455 dead husbands of non-cancer widows, together with the distribution of these deaths as percentages of the deaths in each age group of men who were husbands of non-cancer widows. It also shows the age distribution of the 83 cancer deaths among the total of 417 dead husbands of cancer widows, together with the distribution of these deaths as percentages of the deaths in each age group of men who were husbands of cancer widows.

Table VI compares the age distribution of the cancer cases in the husbands of the cancer widows and the non-cancer widows. They are seen to be similar.

TABLE V.—*Age Distribution of Cancer Deaths Among the Two Groups of Husbands*

| Age groups | Husbands of cancer widows: All husbands | Husbands who died from cancer | Per cent | Husbands of non-cancer widows: All husbands | Husbands who died from cancer | Per cent |
|---|---|---|---|---|---|---|
| 0–54 | 43 | 10 | 23·3 | 38 | 10 | 26·4 |
| 55–64 | 91 | 18 | 19·8 | 93 | 19 | 20·5 |
| 65–74 | 166 | 38 | 22·9 | 169 | 40 | 23·6 |
| 75+ | 117 | 17 | 14·5 | 155 | 25 | 16·2 |
| All ages | 417 | 83 | 20·0 | 455 | 94 | 20·6 |

TABLE VI.—*Age Distribution of Cancer Cases in the Two Groups of Husbands*

| Age groups | Husbands of cancer widows who themselves died of cancer: Number | Per cent | Husbands of non-cancer widows who themselves died of cancer: Number | Per cent |
|---|---|---|---|---|
| 0–54 | 10 | 12·1 | 10 | 10·6 |
| 55–64 | 18 | 21·7 | 19 | 20·2 |
| 65–74 | 38 | 45·8 | 40 | 42·6 |
| 75+ | 17 | 20·4 | 25 | 26·6 |
| All ages | 83 | 100 | 94 | 100 |

(b) *Social class.*—Table VII shows that the distribution of the cancer cases among the social classes follows that of the group of husbands of which they form part and is generally similar in both groups. Nevertheless, a relatively

TABLE VII.—*Social Class Distribution of Cancer Cases in the Two Groups of Husbands*

| | Social Class: 1 | 2 | 3 | 4 | 5 | Not stated | Total |
|---|---|---|---|---|---|---|---|
| **Cancer cases among Husbands of Cancer Widows** | | | | | | | |
| No. | 4 | 12 | 42 | 11 | 12 | 2 | 83 |
| % | 4·8 | 14·6 | 50·4 | 13·3 | 14·5 | 2·4 | 100 |
| **Cancer cases among Husbands of Non-cancer Widows** | | | | | | | |
| No. | 5 | 9 | 48 | 19 | 12 | 1 | 94 |
| % | 5·3 | 9·6 | 51·0 | 20·3 | 12·7 | 1·1 | 100 |

larger number of cases fall into Group 2 and a small number into Group 4 amongst the pairs where both husband and wife died from cancer than amongst the pairs where the husband only died from cancer.

(c) *Geographical distribution of cases of cancer in the husbands.*—Table VIII shows the distribution amongst the 6 areas in the survey of the 83 cases of cancer in the husbands of cancer widows and the percentage in each area. It also shows the distribution amongst the same areas of the 94 cases of cancer in the husbands of non-cancer widows. Comparison of the two groups shows that the percentages were very similar in all areas except Gosport where the numbers were very small. It is also seen that in Banstead the percentage of cancer in both groups, i.e. 31·5 per cent and 33·3 per cent, was higher than the total percentages (20 per cent and 20·6 per cent respectively).

TABLE VIII.—*Numbers and Percentages of Husbands in Each Area who Died From Cancer, for Each of the Two Groups of Widows*

| | Cancer widows | | | Non-cancer widows | | |
|---|---|---|---|---|---|---|
| Districts | Total | With cancer husbands | Per cent | Total | With cancer husbands | Per cent |
| Gosport | 9 | 1 | 11·0 | 13 | 4 | 30·8 |
| Eastleigh | 56 | 14 | 25.0 | 63 | 11 | 17·5 |
| Winchester | 34 | 5 | 14·7 | 32 | 5 | 15·6 |
| Banstead | 38 | 12 | 31·5 | 27 | 9 | 33·3 |
| Merton and Morden | 94 | 21 | 22·3 | 109 | 26 | 23·8 |
| Fulham | 186 | 30 | 16·1 | 211 | 39 | 18·5 |
| All districts | 417 | 83 | 20·0 | 455 | 94 | 20·6 |

*Association of Specific Sites of Neoplasm in Husband and their Wives*

The above remarks apply to cancer of all sites taken together. An examination was also made to see whether the husbands of women dying from growths of particular sites were themselves more likely than might be expected by chance (i.e. more than the husbands of wives who did not die from cancer would be) to die from the same particular site growth, or cancer of some other site. An account of this is given in the Appendix and Table IX.

### DISCUSSION AND COMMENTS ON RESULTS

It will be seen from the above results that the percentage of husbands of cancer widows who also died of cancer did not differ from the percentage of husbands of non-cancer widows who died from cancer. It appears then that the occurrence of cancer in the wives was not linked with the occurrence of cancer in their husbands who pre-deceased them. To put matters simply, if a wife died from cancer there was no more chance that her husband would die from cancer than if he were the husband of someone else who did not die from cancer. Therefore, there is no evidence, from this investigation (at this time and place), that habits common to husband and wife have detectable importance in causation of cancers from which both could suffer.

TABLE IX

| Diagnosis | All cancer widows | Husband and wife with same growth No. | Husband and wife with same growth % | Non-cancer widows' husbands who died from growth of these sites No. | Non-cancer widows' husbands who died from growth of these sites % (of 455) |
|---|---|---|---|---|---|
| Tongue | 1 | — | .. | .. | .. |
| Floor of mouth | 1 | — | .. | .. | .. |
| Other parts of mouth | 1 | — | .. | .. | .. |
| Hypopharynx | 1 | — | .. | .. | .. |
| Oesophagus | 6 | — | .. | .. | .. |
| Stomach | 65 | 3 | 4·6 | 17 | 3·7 |
| Small intestine | 2 | — | .. | .. | .. |
| Large intestine exc. rectum | 49 | 1 | 2·0 | 13 | 2·9 |
| Rectum | 36 | 1 | 2·8 | 10 | 2·2 |
| Extra-hepatic ducts | 14 | — | .. | .. | .. |
| Liver (secondary) | 3 | — | .. | .. | .. |
| Pancreas | 13 | — | .. | .. | .. |
| Peritoneum | 5 | — | .. | .. | .. |
| Larynx | 2 | — | .. | .. | .. |
| Trachea, bronchus and lung | 19 | 2 | 10·0 | 20 | 4·3 |
| Mediastinum | 2 | — | .. | .. | .. |
| Breast | 77 | — | .. | .. | .. |
| Cervix uteri | 20 | — | .. | .. | .. |
| Corpus uteri | 5 | — | .. | .. | .. |
| Uterus, unspecified | 14 | — | .. | .. | .. |
| Ovary and fallopian tube | 21 | — | .. | .. | .. |
| Unspecified fem. gen. organs | 2 | — | .. | .. | .. |
| Kidney | 4 | — | .. | .. | .. |
| Bladder and urin. organs | 11 | — | .. | .. | .. |
| Malignant melanoma of skin | 1 | — | .. | .. | .. |
| Other malignant neoplasm of skin | 3 | — | .. | .. | .. |
| Eye | 1 | — | .. | .. | .. |
| Brain and other parts of N.S. | 4 | — | .. | .. | .. |
| Thyroid gland | 2 | — | .. | .. | .. |
| Bone (including jaw bone) | 3 | — | .. | .. | .. |
| Secondary and unspec. malig. neoplasm of lymph nodes | 1 | — | .. | .. | .. |
| Other and unspecified sites | 16 | — | .. | .. | .. |
| Lymphosarcoma and reticulosarcoma | 2 | — | .. | .. | .. |
| Hodgkin's disease | 3 | — | .. | .. | .. |
| Lymphatic leukaemia | 1 | — | .. | .. | .. |
| Myeloid leukaemia | 2 | — | .. | .. | .. |
| Acute leukaemia, type unspecified | 4 | — | .. | .. | .. |
| | 417 | 7 | .. | .. | .. |

N.B.—To reduce the complexity of presentation the detailed site distribution of all cancer deaths in the two groups of husbands is omitted from this Table. It is available on request.

This conclusion is not invalidated by the fact that we cannot draw up a hard and fast list of habits that are or are not always either individual or common to husband and wife.

The above remarks are likely to be true even if we assume that a complex of factors in heredity and environment (both inside and outside the home) is needed to set the stage necessary for a particular growth to occur.

Can our negative result be interpreted as meaning that, in future endemiological investigations based on questionnaires, we can exclude inquiry into domestic habits usually common to members of the same household? It might; but because this could lead to neglect of a large field of inquiry it would be safer to say " The negative result suggests that an investigation of domestic habits (in relation to cancer aetiology) would be more likely to be profitable if directed towards habits usually peculiar to individuals in a household than towards those common to most members of the household ". Nevertheless, although this wide, negative finding might guide future investigations, it should not limit their field, lest some important fact should, by neglect, fail to be revealed.

### CONCLUSION

There is no evidence, from the present investigation, to confirm the hypothesis that domestic factors or habits common to husband and wife are carcinogenic.

The results seem to suggest that it would be less profitable to investigate the possible carcinogenic influence of common domestic factors than other, usually unshared, factors. However, this conclusion should be applied with caution to future plans for endemiological enquiries lest a conceivably important field for further investigation be neglected.

### SUMMARY

This investigation attempts to answer the question " How important are shared domestic environmental factors in the aetiology of cancers?". To eliminate family genetic factors a study was made of the causes of death of husbands and wives.

The material consisted of 417 widows who died from cancer (" cancer widows ") and their husbands, the causes of all the deaths being known. A carefully matched control group of 455 widows who did not die from cancer (non-cancer widows) together with their husbands, the causes of whose deaths were also known, was used for comparison. The cancer widows and control widows were closely comparable in respect of age, social class, place of residence, date of death, etc.

It was found that 20 per cent of the husbands of cancer widows died from cancer. The percentage of husbands of non-cancer widows who died from cancer was 20·6 per cent.

It is concluded that, as cancer in the husbands of women who died from cancer was no more frequent than cancer in the husbands of women who did not die from cancer, then it is unlikely that shared domestic environmental factors are important carcinogenic agents for the time, places and people in this study.

An appendix describes the observed percentage of cancers of identical sites in cancer widows and their husbands and compares these with the findings in the group of non-cancer-widows and their husbands.

### *NOTE*

When this paper was written the literature was searched under the heading " Cancer in Husbands and Wives " without result. Because the author happened to come across a reference to Ciocco's work the literature was re-searched under the heading " Mortality in Husbands and Wives ". This revealed Ciocco's (1940, 1941, 1942) papers. Using an approach similar but not identical to the method described in the present paper Ciocco found an excess of cancer above expectation in the spouses of cancer subjects, i.e. his results are the reverse of our own.

Evelyn A. Potter and Mildred R. Tully, deliberately following Ciocco's methods as closely as they could in Massachusetts, failed to confirm his results. Their findings, then, are compatible with our own, though they used a somewhat different method.

Obviously from the above conflicting results further investigations are required and until these have been carried out, generalisations about the presence or absence of association of cancers in husbands and wives are unjustifiable.

Thanks are due to the following Medical Officers of Health for allowing access to their records for this research :

| | |
|---|---|
| Dr. E. J. MacIntyre | (Banstead U.D.C.) |
| Dr. W. Alastair Glen | (Eastleigh M.B.) |
| Dr. M. I. Adams | (Fulham M.B.) |
| Dr. Percival W. Pritchard | (Gosport M.B.) |
| Dr. W. D. Swinney | (Merton and Morden U.D.C.) |
| Dr. Robt. A. Good | (City of Winchester). |

My special thanks are due to Miss Joyce Strudwick and Miss Rosemary Axe, B.A. who carried out most of the investigation. Thanks for co-operation are due to Miss V. P. Farrow and her staff at the South West London Mass X-ray Service, especially to Mrs. V. Collins, Mrs. M. Levett, Mrs. W. E. Smith, Miss A. Taylor, Mrs. V. Thomas and Miss K. E. Janvrin Tims.

I am also grateful to the Librarian of the Royal Society of Medicine for searching the literature.

I am required to state that this investigation formed part of a successful entry for the South West Metropolitan Regional Hospital Board Research Prize Competition 1958.

### REFERENCES

Ciocci, A.—(1940) *Proc. nat. Acad. Sci., Wash.*, **26**, 610–5. (Prelim. note to art. in *Hum. Biol.*)—(1940) *Hum. Biol.*, **12**, 508–31.—(1941) *Ibid.*, **13**, 189–202 (May).—(1942) *Publ. Hlth Rep.*, **57**, 1333–42 (Sep. 4).

Potter, E. A. and Tully, M. R.—(1945) *Amer. J. publ. Hlth.*, **35**, 485–90 (May).

### APPENDIX

Table IX shows the type occurrence of the 417 cancers occurring in the cancer widows (col. 1). Column 2 of this table shows the number of deaths from cancer of the same site occurring among the husbands of women dying from cancer of that particular site.

Of 65 women who died from carcinoma of the stomach, 15 (23 per cent) had husbands who died from cancer of which 3 died from cancer of the stomach, i.e. 4·6 per cent, whereas of the 455 women not dying from cancer, 17 had husbands die from stomach cancer (3·7 per cent) and of the 352 women who died from cancer of other sites the husbands of 10 died from cancer of the stomach (2·8 per cent).

Of 49 women who died from carcinoma of the colon, 8 (16·4 per cent had husbands who died from cancer of which 1 died from cancer of the colon, i.e. 2 per cent whereas of the 455 women not dying from cancer 13 had husbands die from carcinoma of the colon (2·9 per cent) and of the 368 women who died from cancer of other sites the husbands of 7 died from cancer of the colon (1·9 per cent).

Of 36 women who died from carcinoma of the rectum, 8 (22 per cent) had husbands who died from cancer of which 1 died from cancer of the rectum, i.e. 2·8 per cent whereas of the 455 women not dying from cancer 10 had husbands die from cancer of the rectum (2·2 per cent), and of the 381 women who died from. cancer of other sites the husbands of 7 died from cancer of the rectum (1·8 per cent).

Of 19 women who died from lung cancer 5 (26·3 per cent) had husbands also die from cancer of which 2 died from lung cancer, i.e. 10 per cent, whereas of 455 women who died from causes other than cancer, 20 had husbands die from lung cancer (4·3 per cent), and of the 398 women who died from cancer of other sites the husbands of 17 died from lung cancer (4·4 per cent).

We have already described the absence of unexpected association of the same cancer in husband and wife in respect of the following sites :— stomach, colon, rectum and lung. Looking down the list of remaining sites no example is seen of the same growth occurring in husband and wife among the less common growths.

Examination of the series for evidence of associated causes of death in husbands and wives other than malignancy was undertaken but will not be described here, the number of diseases being so large in proportion to the number of deaths available that no reliable conclusions are possible. For the same reason it was not possible to make a reliable investigation into the possibility of an association between the occurrence of a particular site of cancer in the wife and some other specific non-malignant disease in the husband.